# Absent words and the (dis)similarity analysis of DNA sequences: an experimental study

**DOI:** 10.1186/s13104-016-1972-z

**Published:** 2016-03-22

**Authors:** Mohammad Saifur Rahman, Ali Alatabbi, Tanver Athar, Maxime Crochemore, M. Sohel Rahman

**Affiliations:** Department of CSE, AlEDA Group, BUET, West Palasi, Dhaka, 1205 Bangladesh; Department of Informatics, King’s College London, Strand, London, UK; Université Paris-Est, Créteil Cedex, France

**Keywords:** Absent words, Minimal absent words, Alignment free comparison, Distance matrix, Phylogenetics

## Abstract

**Background:**

An absent word with respect to a sequence is a word that does not occur in the sequence as a factor; an absent word is minimal if all its factors on the other hand occur in that sequence. In this paper we explore the idea of using minimal absent words (MAW) to compute the distance between two biological sequences. The motivation and rationale of our work comes from the potential advantage of being able to extract as little information as possible from large genomic sequences to reach the goal of comparing sequences in an alignment-free manner.

**Findings:**

We report an experimental study on the use of absent words as a distance measure among biological sequences. We provide recommendations to use the best index based on our analysis. In particular, our analysis reveals that the best performers are: the length weighted index of relative absent word sets, the length weighted index of the symmetric difference of the MAW sets, and the Jaccard distance between the MAW sets. We also found that during the computation of the absent words, the reverse complements of the sequences should also be considered.

**Conclusion:**

The use of MAW to compute the distance between two biological sequences has potential advantage over alignment based methods. It is expected that this potential advantage would encourage researchers and practitioners to use this as a (dis)similarity measure in the context of sequence comparison and phylogeny reconstruction. Therefore, we present here a comparison among different possible models and indexes and pave the path for the biologists and researchers to choose an appropriate model for such comparisons.

**Electronic supplementary material:**

The online version of this article (doi:10.1186/s13104-016-1972-z) contains supplementary material, which is available to authorized users.

## Findings

### Background

Recently, the concept of minimal absent word (MAW) has been used to compute the distance between two species [1]. Similar effort has also been made to investigate the variation in number and content of MAWs within a species using four human genome assemblies [2]. This concept along with the related notions of absent words, also known as nullomers and forbidden words, have received significant attention in the relevant literature (e.g., [3–11]) and have been shown to be useful in applications like text compression [12,13]. Perhaps the most significant use of this concept is in the field of computational biology. Hampikian and Andersen have studied nullomers, i.e., the shortest words that do not occur in a given genome, and primes, i.e., the shortest words that are absent from the entire known genetic data with a motivation to discover the constraints on natural DNA and protein sequences [14]. Acquisti et al. [15] have studied nullomers and the cause of absent words in the human genome. Herold et al. [16] have presented a method to compute the shortest absent words in genomic sequences. Pinho et al. [17] on the other hand focused on MAWs that form a set smaller than the set of absent words. Subsequently, Garcia and Pinho have studied four human genome assemblies from the perspective of MAWs [2].

The main focus of this paper is to study and analyze possible indexes that can be used with MAWs to establish an alignment-free distance or similarity measure. The motivation and rationale of using MAW comes from the potential advantage of being able to extract as little information as possible from large genomic sequences to reach the goal of comparing them with one another. And this has recently attracted researchers to propose distance measures based on MAWs. For example, in [1], Chairungsee and Crochemore have proposed a distance measure based on the set of MAWs and have used that distance measure to construct a phylogenetic tree among 11 species, following an experimental setup of Liu and Wang [18]. And, in [2], Garcia and Pinho have explored the potential of the MAWs from the perspective of similarities and differences among 4 human genome assemblies.

While the use of MAW set as a distance measure seems interesting and useful, to the best of our knowledge there exists no attempt in the literature to identify the best index to employ on the MAW set. Indeed, Chairungsee and Crochemore [1] chose to employ Length-weighted index (LWI) on the symmetric difference of two MAW sets but without any discussion on the motivation and rationale behind their choice. While it is likely that the potential advantage of MAW set would encourage researchers and practitioners to use this as a (dis)similarity measure in the context of sequence comparison and phylogeny reconstruction, the lack of any directions on which index to use with it may remain as an obstacle. This is where our current research work fits in. In this work we conduct an experimental study on the same setting of [18] and [1] to analyze and identify the best index to use the MAWs as a distance/similarity measure. In our experiments we have analyzed all the index/matrices that are already used in the literature. Additionally we have used some well-studied indexes for the first time as a distance measure using MAWs. Table 1 lists and comments on the indexes considered in this paper. In the sequel, based on our analysis and comparison among the different methods studied, we have presented some recommendations with a goal to aid the researchers to select a suitable method for such similarity/dissimilarity analysis.

**Table 1:** Indexes used and compared in this paper as a distance/similarity measure

Index	Comment
Length-weighted index (LWI)	Considered in [1] for only symmetric difference. Here we also use it for set intersection
Jaccard distance	Used in this paper
Total variation distance (TVD)	Used in [2] to analyze similarity on four human genome assemblies
GC content	Used in [2] to analyze similarity on four human genome assemblies. Here we use GC content on symmetric difference, set intersection of MAW sets as well as on RAW sets
Relative absent word (RAW)	Considered in [20] to study Ebola virus genomes against human DNA. Here we use RAW sets for LWI and GC content measures

### Methods

A string $x = x_1, x_2, \ldots , x_n$ is a sequence of characters of length *n* from a finite alphabet $\Sigma$, i.e., $x_i \in \Sigma , 1\le i\le n$. An empty string is denoted by $\epsilon$. A string *y* is a factor or substring of a string *x* iff there exist strings *u*, *v* such that $x=uyv$; if $u\ne \epsilon$ or $v\ne \epsilon$, then, *y* is a proper factor of *x*. We use the term *word* and *string* synonymously. An absent word in a string is a word that does not occur in the given string. More formally, a string *y* is an absent word in a string *x* if it is not a factor of *x*. Additionally, if all its proper factors are factors of *x*, then *y* is said to be a MAW. For example, *aaa*, *aba*, and *bbb* are examples of MAWs for the string $x = abbaab$. But, *aaab* is an absent word but not a MAW of *x*. Given a string *x*, we will use $MAW_x$ to denote the set of MAWs of *x*.

Given a set, $\mathcal S = \{s_1, s_2, \ldots , s_k\}$ of *k* sequences, we employ the following methodology:

Step 1: For each sequence $s_i, 1\le i\le k$, we compute $MAW_{s_i}$.
Step 2: We compute distance matrix $\mathcal M_{\mathcal S}^{\mathcal D}$ for the set $\mathcal S$ using a distance measure $\mathcal D$ based on $MAW_{s_i}, 1\le i\le k$. For all $1\le i,j\le k$, we have $\mathcal M_{\mathcal S}^{\mathcal D}[i,j] = \mathcal D[s_i, s_j]$. Because the distance measure is symmetric, we need only focus on the upper triangle of the matrix $\mathcal M_{\mathcal S}^{\mathcal D}$.
Step 3: We build a phylogenetic tree $\mathcal T^{\mathcal D}_{\mathcal A}(\mathcal S)$ on the set $\mathcal S$ based on the distance measure $\mathcal D$ applying algorithm $\mathcal A$ on $\mathcal M_{\mathcal S}^{\mathcal D}$ for phylogeny reconstruction.

#### Distance measures

We apply a number of distance measures discussed below. In what follows we will consider two sequences *x* and *y* and their MAW sets, $MAW_{x}$ and $MAW_{y}$.

##### Length-weighted index

In [1], the LWI has been studied and experimented. There, this measure has been applied on the symmetric difference of the MAW sets. In our study we apply intersection operation as well. Formally:

1 $$\begin{aligned} LWI_\Delta (x,y) = \sum _{u\in MAW_{x}\Delta MAW_{y} } \frac{1}{|u|^2} \end{aligned}$$

2 $$\begin{aligned} LWI_{\bigcap }(x,y) = - \sum _{u\in MAW_{x}\bigcap MAW_{y} } \frac{1}{|u|^2} \end{aligned}$$

Here, $\Delta$ and $\bigcap$ refer to the set symmetric difference and set intersection operations. Note that, the intersection operation between two sets can be seen as a similarity measure and hence we use negation in Eq. 2.

##### Jaccard distance

Jaccard index is a statistical measure to use as a similarity coefficient between sample sets. Because we are interested in a distance matrix we use the following equation (based on Jaccard index) for computing the Jaccard distance.

3 $$\begin{aligned} J(x,y) = 1- \frac{\left| MAW_{x}\bigcap MAW_{y}\right| }{\left| MAW_{x}\bigcup MAW_{y}\right| } \end{aligned}$$

##### Total variation distance (TVD)

Garcia and Pinho [2] used TVD to assess pairwise variance. The definition of TVD is as follows:

4 $$\begin{aligned} TVD(P,Q) = \frac{1}{2}\sum _{i}\left| P(i) - Q(i)\right| , \end{aligned}$$

where *P* and *Q* are two probability measures over a finite alphabet, and the term 1 / 2 corresponds to the normalization by the two probability distributions [19]. This distance measure has values in the interval [0, 1] with higher values implying greater dissimilarity or difference. To calculate *TVD* (*x*, *y*), i.e., TVD between two sequences *x* and *y* we first count the number of MAWs in $MAW_x$ and $MAW_y$ for each word size and then transform this histogram in a normalized version that can be interpreted as a probability distribution. Subsequently, TVD is computed according to Eq. 4.

##### *GC* content

The above-mentioned indexes are based on the number statistics of the MAW sets. Inspired by the work of [2], we make an effort to suggest a measure that is more related to the content of the MAWs. In particular we focus on the compositional bias or *GC* content of the MAW sets. The GC content is the overall fraction of *G* plus *C* nucleotides in each set. We compute the GC content considering both symmetric difference and intersection. Assume that $NUM_{\alpha }(P)$ provides the number of a particular character $\alpha \in \Sigma$ in the members of the set *P* and $NUM_{\Sigma }(P)$ provides the number of all characters in the members of the set *P*. Then, formally:

5 $$\begin{aligned} GCC_\Delta (x,y) = \frac{NUM_G\left( MAW_x \Delta MAW_{y}\right) +NUM_C \left( MAW_x \Delta MAW_{y}\right) }{NUM_\Sigma \left( MAW_x \Delta MAW_{y}\right) } \end{aligned}$$

6 $$\begin{aligned} GCC_{\bigcap }(x,y) = 1 - \frac{NUM_G \left( MAW_x \bigcap MAW_{y}\right) +NUM_C \left( MAW_x \bigcap MAW_{y}\right) }{NUM_\Sigma \left( MAW_x \bigcap MAW_{y}\right) } \end{aligned}$$

#### Relative absent words (RAWs)

Very recently, Silva et al. [20] have conducted a study on Ebola virus genomes against human DNA where they have applied a new concept called the RAW. RAW has been defined in [20] in the context of a target sequence *x* and a reference sequence *y*. Suppose $W_k(x) ~\left( \overline{W_k(x)}\right)$ denotes the set of all *k*-length factors of (that are not present in) *x*. So, $R_k(x,\overline{y})$ denotes the set of all words that exist in *x* but do not exist in *y*:

7 $$\begin{aligned} R_k(x,\overline{y}) = W_k(x) \bigcap \overline{W_k(y)} \end{aligned}$$

Now, we are interested in the subset of words that are minimal in the sense the MAWs are defined. Because a minimal absent word of size *k* cannot contain any MAW of size less than *k*, we can have the following definition for RAWs:

8 $$\begin{aligned} M_k(x,\overline{y}) = \{\alpha \in R_k(x,\overline{y})~:~W_{k-1}(\alpha )\bigcap M_{k-1}(x,\overline{y})=\emptyset \} \end{aligned}$$

Now, Silva et al. [20] used RAW for differential identification of sequences that are derived from a pathogen genome (i.e., EBOLA virus) but absent from its host (i.e., human). This inspires us to use RAW to compute the distance between two species in our study. Here we have used their software called EAGLE to compute the set of RAWs considering each species in turn as the reference and the remaining species as targets. To elaborate, recall that we were given a set, $\mathcal S = \{s_1, s_2, \ldots , s_k\}$ of *k* sequences. For a particular pair of sequence $s_i, s_j \in \mathcal S$, we first compute $RAW_{s_i,s_j}~(RAW_{s_j,s_i})$, i.e., the set of RAWs considering $s_i~(s_j)$ as the reference and $s_j~(s_i)$ as the target sequence. Then we compute the Length Weighted Index (LWI) (discussed above) of both $RAW_{s_i,s_j}$ and $RAW_{s_j,s_i}$. This gives us two distance values for a particular pair of species. We then take the average of these two distance measures. Similarly, we also apply the *GC* content measure on the RAW sets.

### Results and discussion

We have used the same datasets used in [18] and [1]. In particular, we have conducted our experiments on the first exon sequences of $\beta$-globin genes from 11 species, namely, Human, Goat, Gallus, Opossum, Lemur, Mouse, Rabbit, Rat, Bovine, Gorilla, and Chimpanzee. Because the gene family of $\beta$-globin has a significant biological role in oxygen transport in organisms, it is used to analyze DNA and the first exon of the $\beta$-globin gene is an example for many DNA studies instead of computing similarity/dissimilarity of the whole genomes. Inspired by the experimental setup of Garcia and Pinho [2], we consider two scenarios: the original sequence itself and the original sequence concatenated with its reversed complement (artificial words across the boundary between both sequences are ignored). The former will be referred to as the noRC setting and the latter as the RC setting. The motivation for using the reverse complement is to take into consideration words that might occur in the reverse complement strand but that might be absent from the direct strand.

We have used the algorithm of [11] to compute the MAW sets using their implementation, which is available at: http://github.com/solonas13/maw. We have used EAGLE software of [20] to compute the RAW sets; EAGLE is available at: http://bioinformatics.ua.pt/software/eagle/. The code to compute the distance matrices and analyze the results were written in C++ language and can be found at: https://github.com/srautonu/AWorDS. We have also implemented a related web-based tool with limited capacity here: http://www.ekngine.com/AWorDS. It is planned that this web-tool will be improved with more functionalities in near future.

We have considered five distance measures described in “Distance measures” section based on the MAW sets. Additionally, we have considered LWI and *GC* content distance measures involving RAW sets. With noRC and RC settings, this gives us a total of 14 distance matrices. For the sake of brevity we do no provide all the distance matrices in this paper. However, these can be found here: https://github.com/srautonu/AWorDS and also in the Additional files 1, 2, 3 and 4.

#### Discussion

Following the methodology of [18] we have carefully analyzed the computed distance matrices based on the real biological phenomena that are also considered in [18]:

- It is believed that Gorilla and Chimpanzee are most similar to Human [REL 1];
- Similarly, among these 11 species, Goat and Bovine should be similar [REL 2] as are Rat and Mouse [REL 3];
- Gallus and Opossum should be remote from the other species because Gallus is the only non-mammalian representative in this group [REL 4] and Opossum is the most remote species from the remaining mammals [REL 5];
- Besides gallus and Opossum, lemur is more remote from the other species relatively [REL 6].

We have analyzed the distance measures based on the above-mentioned six expected relations (REL 1–REL 6). Among these six relations we give higher importance on REL 1 through REL 3 in the sense that when all of these are captured we look into the rest for further comparison. Below we discuss several interesting points from our analysis. Notably, we have provided a spreadsheet (Additional file 4) with a brief description of the content as a Additional files 1, 2, 3 and 4 that we have used for this analysis.

- As is evident from our analysis, unfortunately, the GCC measure does not do very well in comparison to the other metrics despite that it is more related to the content of the minimal absent words. In particular, in most cases this measure is unable to capture the expected relationships (REL 1–REL 6) mentioned above. However, despite the overall relative poor performance, except for the cases when intersection operation has been used, GCC measure is at least able to capture the close relation among Human, Gorilla and Chimpanzee, i.e., REL 1. For intersection operation however, GCC fails miserably to capture any of the important relationships among REL 1 REL 2 and REL 3.
- The TVD also fails to be highly impressive. It has been able to capture some of the relations but not all. However, it definitely seems better than the GCC measures. In particular, it has been able to capture REL 1 and in most cases it also captures REL 2. However, it fails to capture REL 3 in both RC and NoRC settings.

**Table 2:** The distance matrix based on the length weighted index on RAW sets (on RC setting)

Species	Human	Goat	Opossum	Gallus	Lemur	Mouse	Rabbit	Rat	Gorilla	Bovine	Chimp
Human		23.39	26.94	28.34	27.82	23.49	19.31	27.88	4.77	21.60	7.26
Goat			28.71	24.16	25.89	25.52	24.33	27.43	21.77	8.73	24.26
Opossum				29.55	31.23	29.21	26.69	30.52	26.90	28.16	28.44
Gallus					28.66	30.22	26.27	30.89	28.25	26.21	30.51
Lemur						30.21	27.63	30.96	27.77	25.91	30.27
Mouse							24.09	26.43	20.98	23.17	23.29
Rabbit								29.19	19.02	22.28	21.50
Rat									28.37	27.95	30.21
Gorilla										19.48	9.62
Bovine											21.97
Chimp

**Table 3:** The sorted list of each species from a particular species (left most column of each row) according to the computed distance based on the length weighted index on RAW sets (on RC setting)

Human	$\rightarrow$Gorilla	$\rightarrow$Chimp	$\rightarrow$Rabbit	$\rightarrow$Bovine	$\rightarrow$Goat	$\rightarrow$Mouse	$\rightarrow$Opossum	$\rightarrow$Lemur	$\rightarrow$Rat	$\rightarrow$Gallus
Goat	$\rightarrow$Bovine	$\rightarrow$Gorilla	$\rightarrow$Human	$\rightarrow$Gallus	$\rightarrow$Chimp	$\rightarrow$Rabbit	$\rightarrow$Mouse	$\rightarrow$Lemur	$\rightarrow$Rat	$\rightarrow$Opossum
Opossum	$\rightarrow$Rabbit	$\rightarrow$Gorilla	$\rightarrow$Human	$\rightarrow$Bovine	$\rightarrow$Chimp	$\rightarrow$Goat	$\rightarrow$Mouse	$\rightarrow$Gallus	$\rightarrow$Rat	$\rightarrow$Lemur
Gallus	$\rightarrow$Goat	$\rightarrow$Bovine	$\rightarrow$Rabbit	$\rightarrow$Gorilla	$\rightarrow$Human	$\rightarrow$Lemur	$\rightarrow$Opossum	$\rightarrow$Mouse	$\rightarrow$Chimp	$\rightarrow$Rat
Lemur	$\rightarrow$Goat	$\rightarrow$Bovine	$\rightarrow$Rabbit	$\rightarrow$Gorilla	$\rightarrow$Human	$\rightarrow$Gallus	$\rightarrow$Mouse	$\rightarrow$Chimp	$\rightarrow$Rat	$\rightarrow$Opossum
Mouse	$\rightarrow$Gorilla	$\rightarrow$Bovine	$\rightarrow$Chimp	$\rightarrow$Human	$\rightarrow$Rabbit	$\rightarrow$Goat	$\rightarrow$Rat	$\rightarrow$Opossum	$\rightarrow$Lemur	$\rightarrow$Gallus
Rabbit	$\rightarrow$Gorilla	$\rightarrow$Human	$\rightarrow$Chimp	$\rightarrow$Bovine	$\rightarrow$Mouse	$\rightarrow$Goat	$\rightarrow$Gallus	$\rightarrow$Opossum	$\rightarrow$Lemur	$\rightarrow$Rat
Rat	$\rightarrow$Mouse	$\rightarrow$Goat	$\rightarrow$Human	$\rightarrow$Bovine	$\rightarrow$Gorilla	$\rightarrow$Rabbit	$\rightarrow$Chimp	$\rightarrow$Opossum	$\rightarrow$Gallus	$\rightarrow$Lemur
Gorilla	$\rightarrow$Human	$\rightarrow$Chimp	$\rightarrow$Rabbit	$\rightarrow$Bovine	$\rightarrow$Mouse	$\rightarrow$Goat	$\rightarrow$Opossum	$\rightarrow$Lemur	$\rightarrow$Gallus	$\rightarrow$Rat
Bovine	$\rightarrow$Goat	$\rightarrow$Gorilla	$\rightarrow$Human	$\rightarrow$Chimp	$\rightarrow$Rabbit	$\rightarrow$Mouse	$\rightarrow$Lemur	$\rightarrow$Gallus	$\rightarrow$Rat	$\rightarrow$Opossum
Chimp	$\rightarrow$Human	$\rightarrow$Gorilla	$\rightarrow$Rabbit	$\rightarrow$Bovine	$\rightarrow$Mouse	$\rightarrow$Goat	$\rightarrow$Opossum	$\rightarrow$Rat	$\rightarrow$Lemur	$\rightarrow$Gallus

- Among the distance measures one of the best (if not the best) performers turns out to be the length weighted index applied on the RAW sets. In particular, Table 2 (also see Table 3) has all the desired relations (REL 1 through REL 6) mentioned above. As expected, the result is better when RC setting is used.

**Table 4:** The distance matrix based on the Jaccard distance on MAW sets (on RC setting)

Species	Human	Goat	Opossum	Gallus	Lemur	Mouse	Rabbit	Rat	Gorilla	Bovine	Chimp
Human		0.70	0.82	0.80	0.76	0.70	0.61	0.80	0.15	0.69	0.26
Goat			0.84	0.74	0.74	0.77	0.77	0.79	0.69	0.36	0.71
Opossum				0.85	0.87	0.91	0.84	0.90	0.82	0.85	0.82
Gallus					0.81	0.82	0.79	0.85	0.80	0.81	0.80
Lemur						0.83	0.81	0.81	0.76	0.72	0.77
Mouse							0.78	0.78	0.64	0.74	0.68
Rabbit								0.81	0.63	0.75	0.65
Rat									0.80	0.82	0.82
Gorilla										0.67	0.15
Bovine											0.69
Chimp

**Table 5:** The sorted list of each species from a particular species (left most column of each row) according to the computed distance based on the Jaccard distance on MAW sets (on RC setting)

Human	$\rightarrow$Gorilla	$\rightarrow$Chimp	$\rightarrow$Rabbit	$\rightarrow$Bovine	$\rightarrow$Mouse	$\rightarrow$Goat	$\rightarrow$Lemur	$\rightarrow$Gallus	$\rightarrow$Rat	$\rightarrow$Opossum
Goat	$\rightarrow$Bovine	$\rightarrow$Gorilla	$\rightarrow$Human	$\rightarrow$Chimp	$\rightarrow$Lemur	$\rightarrow$Gallus	$\rightarrow$Rabbit	$\rightarrow$Mouse	$\rightarrow$Rat	$\rightarrow$Opossum
Opossum	$\rightarrow$Chimp	$\rightarrow$Human	$\rightarrow$Gorilla	$\rightarrow$Rabbit	$\rightarrow$Goat	$\rightarrow$Gallus	$\rightarrow$Bovine	$\rightarrow$Lemur	$\rightarrow$Rat	$\rightarrow$Mouse
Gallus	$\rightarrow$Goat	$\rightarrow$Rabbit	$\rightarrow$Human	$\rightarrow$Gorilla	$\rightarrow$Chimp	$\rightarrow$Bovine	$\rightarrow$Lemur	$\rightarrow$Mouse	$\rightarrow$Opossum	$\rightarrow$Rat
Lemur	$\rightarrow$Bovine	$\rightarrow$Goat	$\rightarrow$Gorilla	$\rightarrow$Human	$\rightarrow$Chimp	$\rightarrow$Rabbit	$\rightarrow$Rat	$\rightarrow$Gallus	$\rightarrow$Mouse	$\rightarrow$Opossum
Mouse	$\rightarrow$Gorilla	$\rightarrow$Chimp	$\rightarrow$Human	$\rightarrow$Bovine	$\rightarrow$Goat	$\rightarrow$Rat	$\rightarrow$Rabbit	$\rightarrow$Gallus	$\rightarrow$Lemur	$\rightarrow$Opossum
Rabbit	$\rightarrow$Human	$\rightarrow$Gorilla	$\rightarrow$Chimp	$\rightarrow$Bovine	$\rightarrow$Goat	$\rightarrow$Mouse	$\rightarrow$Gallus	$\rightarrow$Lemur	$\rightarrow$Rat	$\rightarrow$Opossum
Rat	$\rightarrow$Mouse	$\rightarrow$Goat	$\rightarrow$Human	$\rightarrow$Gorilla	$\rightarrow$Rabbit	$\rightarrow$Lemur	$\rightarrow$Chimp	$\rightarrow$Bovine	$\rightarrow$Gallus	$\rightarrow$Opossum
Gorilla	$\rightarrow$Human	$\rightarrow$Chimp	$\rightarrow$Rabbit	$\rightarrow$Mouse	$\rightarrow$Bovine	$\rightarrow$Goat	$\rightarrow$Lemur	$\rightarrow$Gallus	$\rightarrow$Rat	$\rightarrow$Opossum
Bovine	$\rightarrow$Goat	$\rightarrow$Gorilla	$\rightarrow$Human	$\rightarrow$Chimp	$\rightarrow$Lemur	$\rightarrow$Mouse	$\rightarrow$Rabbit	$\rightarrow$Gallus	$\rightarrow$Rat	$\rightarrow$Opossum
Chimp	$\rightarrow$Gorilla	$\rightarrow$Human	$\rightarrow$Rabbit	$\rightarrow$Mouse	$\rightarrow$Bovine	$\rightarrow$Goat	$\rightarrow$Lemur	$\rightarrow$Gallus	$\rightarrow$Opossum	$\rightarrow$Rat

- Jaccard distance has also turned out to be a very good measure in our experiments. In particular, in Table 4 (also see Table 5) we can identify almost all desired relations (REL 1 through REL 6).
- Length Weighted Index (LWI) for symmetric difference under the RC setting also performs very well in conserving relations REL 1 through REL 5. This measure seems quite good under the NoRC setting as well. However, it is worth-mentioning that under the latter setting it fails to capture the close relation between Rat and Mouse (REL 3).
- In general it seems that the results are better for the RC setting which is expected because this setting takes into consideration words that might occur in the reverse complement strand but that might be absent from the direct strand.

#### Phylogenetic tree reconstruction

**Fig. 1:**
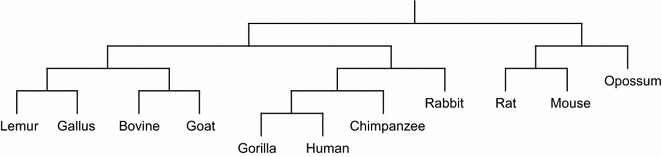
The phylogenetic tree of the 11 species computed using Neighbor Joining algorithm applied on the distance matrix computed based on the length weighted index on the RAW sets (on RC setting)

**Fig. 2:**
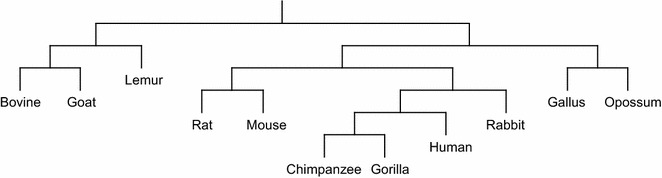
The phylogenetic tree of the 11 species computed using Neighbor Joining algorithm applied on the distance matrix computed based on the length weighted index on symmetric difference of the MAW sets (on RC setting)

**Fig. 3:**
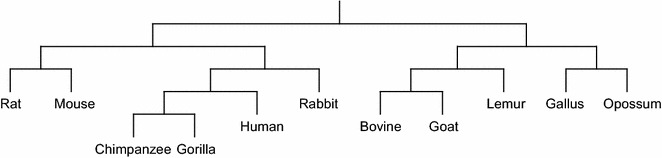
The phylogenetic tree of the 11 species computed using Neighbor Joining algorithm applied on the distance matrix computed based on the Jaccard distance on the MAW sets (on RC setting)

**Fig. 4:**
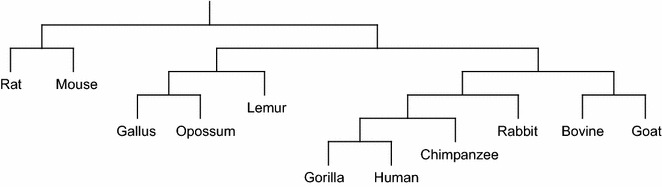
The phylogenetic tree of the 11 species computed using Neighbor Joining algorithm applied on the distance matrix of [18]

Phylogenetic tree of a group of species (taxa) describes the evolutionary relationship among the species. In sequence-based Phylogenetic reconstruction, the input is a set of homologous sequences from different species and these methods construct quite accurate trees on small to moderate sized datasets. Distance based phylogeny reconstruction methods start by computing a matrix that gives us the pairwise *distances* between the sequences under consideration. This distance matrix is then used to estimate the tree using standard clustering methods or specially tailored methods to reconstruct the phylogeny from the distance matrix. The distance measures analyzed in this paper have also been used to reconstruct phylogenetic trees using two well-known methods, namely, unweighted pair group method with arithmetic mean (UPGMA) [21] and Neighbor Joining (NJ) [22]. All the reconstructed phylogenetic trees are presented in Additional files 1, 2, 3 and 4. Here we only present the phylogenetic trees reconstructed using NJ algorithm applied on the distance matrix computed based on the LWI on the RAW sets (Fig. 1), the length weighted index of the symmetric difference of the MAW sets (Fig. 2) and the Jaccard distance (Fig. 3) considering RC setting. Notably, these three indexes are the best performers according to our analysis. Finally, in Fig. 4 we present the phylogenetic tree constructed using NJ algorithm on the distance measure based on Lempel-Ziv complexity proposed in [18] for a visual comparison.

#### Recommendations

In this paper we have experimentally studied a number of distance measures based on the concept of absent words to analyze the similarity/dissimilarity of different sequences. Our main motivation has been to make an experimental study on these so as to provide the community an alignment free method that performs well. Our work is inspired by the previous work with similar goals as in [1] and [18]. In the sequel we present a comparison among the different methods we have studied with a goal to aid the researchers to select a suitable method for such similarity/dissimilarity analysis and phylogeny reconstruction. Based on our analysis we can make the following recommendations:

- LWI applied on the RAW sets, the same, i.e., LWI applied on symmetric difference and Jaccard distance are the best performers and should be used in computing distance matrixes based on absent words.
- RC setting should be preferable. This is supported by the natural assumption that this setting takes into consideration words that might occur in the reverse complement strand but that might be absent from the direct strand.

## Availability of supporting data

The data used in our experiments, the code to compute the distance matrices and analyze the results can be found here: https://github.com/srautonu/AWorDS. The implementation of the algorithm of [11] is available here: http://github.com/solonas13/maw. The EAGLE software of [20] to compute the RAW sets is available here: http://bioinformatics.ua.pt/software/eagle/. We have also setup a preliminary version of a web-based tool here: http://www.ekngine.com/AWorDS.

## Additional files

10.1186/s13104-016-1972-z All Distance Matrices. In this file (AllMatrices), all the distance matrices are provided.
10.1186/s13104-016-1972-z All sorted difference tables. In this file (AllTables), for each distance matrix, a sorted list of each species from a particular species (left most column of each row) is provided.
10.1186/s13104-016-1972-z All phylogenetic trees. In this file (AllTree), all Phylogenetic trees computed based on the distance matrixes are provide. From each distance matrix, teo Phylogenetic trees are reconstructed using two algorithms, namely, UPGMA and NJ.
10.1186/s13104-016-1972-z A Spread-sheet showing scores. In this file (observations.xlsx), for the sake of ease in our analysis, we have computed two scores based on the expected relationships. In all cases, whether the relation exists or not has been checked through visual inspection.
