# Supplementary material for: Absent words and the (dis)similarity analysis of DNA sequences: an experimental study
Source: BMC Res Notes. 2016 Mar 22;9:186. doi: 10.1186/s13104-016-1972-z (PMC4804535; doi:10.1186/s13104-016-1972-z)
Supplement: Supplementary file 1 — 10.1186/s13104-016-1972-z All Distance Matrices. In this file (AllMatrices), all the distance matrices are provided. [file 13104_2016_1972_MOESM1_ESM.pdf]

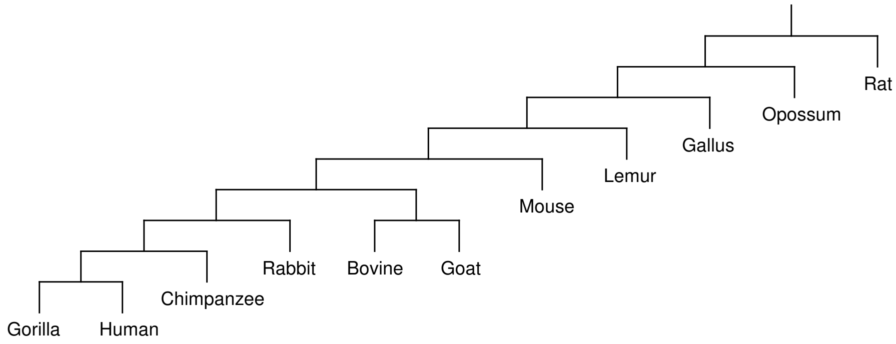

Fig. 1. The phylogenetic tree of the 11 species computed using UPGMA algorithm applied on the distance matrix computed based on the Length Weighted Index on the Symmetric Difference of the MAW sets (on RC setting).

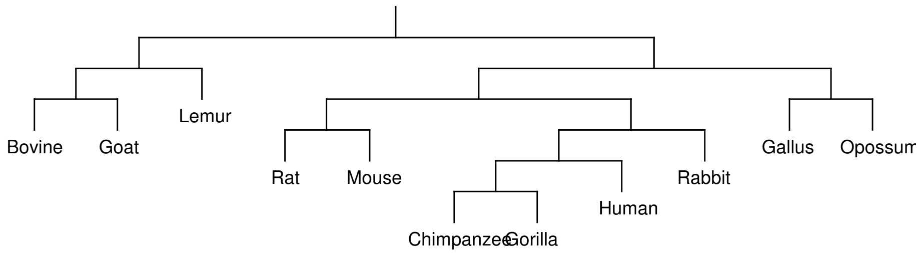

Fig. 2. The phylogenetic tree of the 11 species computed using Neighboring Joining algorithm applied on the distance matrix computed based on the Length Weighted Index on the Symmetric Difference of the MAW sets (on RC setting).

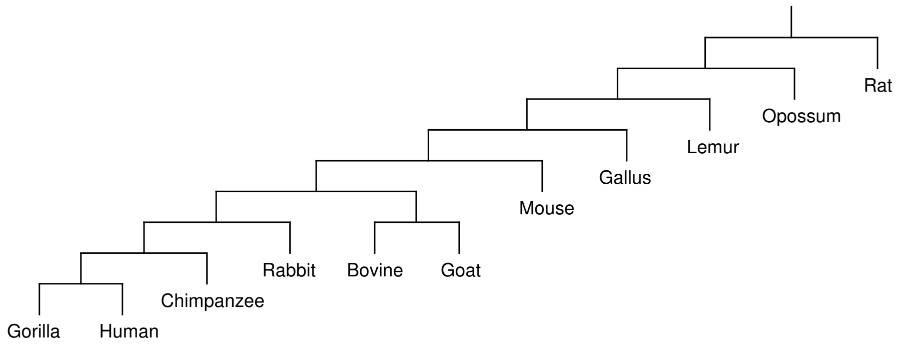

Fig. 3. The phylogenetic tree of the 11 species computed using UPGMA algorithm applied on the distance matrix computed based on the Length Weighted Index on the RAW sets (on RC setting).)

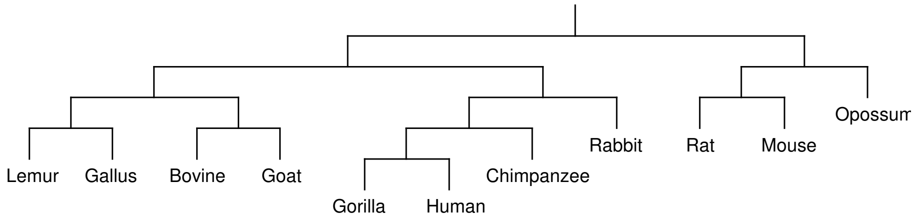

Fig. 4. The phylogenetic tree of the 11 species computed using Neighbor Joining algorithm applied on the distance matrix computed based on the Length Weighted Index on the RAW sets (on RC setting).)

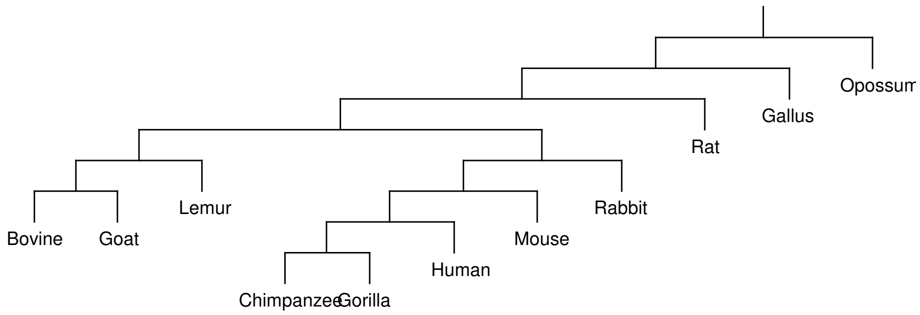

Fig. 5. The phylogenetic tree of the 11 species computed using UPGMA algorithm applied on the distance matrix computed based on the Length Weighted Index on the Intersections of the MAW sets (on RC setting).)

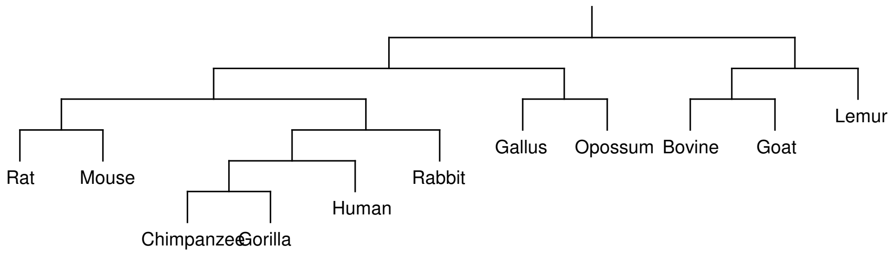

Fig. 6. The phylogenetic tree of the 11 species computed using Neighboring Joining algorithm applied on the distance matrix computed based on the Length Weighted Index on the Intersection of the MAW sets (on RC setting).)

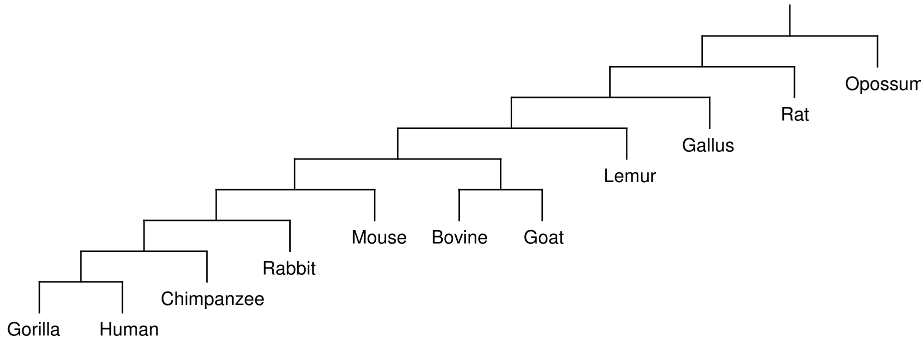

Fig. 7. The phylogenetic tree of the 11 species computed using UPGMA algorithm applied on the distance matrix computed based on the Jaccard Index on the MAW sets (on RC setting).)

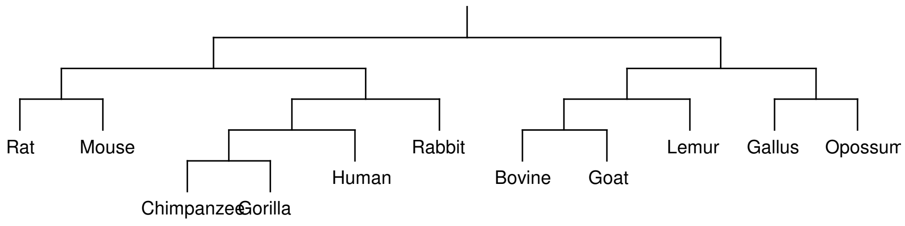

Fig. 8. The phylogenetic tree of the 11 species computed using Neighbor Joining algorithm applied on the distance matrix computed based on the Jaccard Index on the MAW sets (on RC setting).)

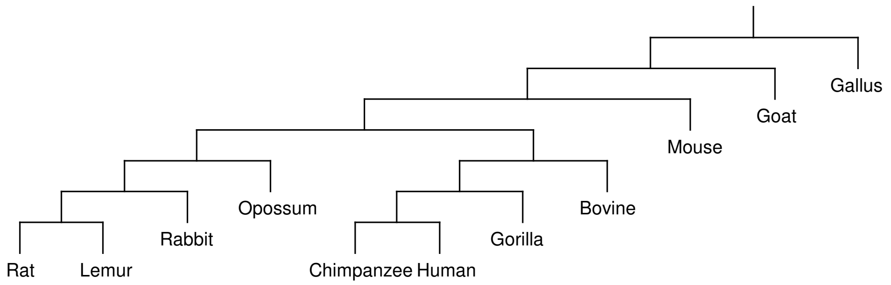

Fig. 9. The phylogenetic tree of the 11 species computed using UPGMA algorithm applied on the distance matrix computed based on the *GC* content on the Symmetric Difference of the MAW sets (on RC setting).)

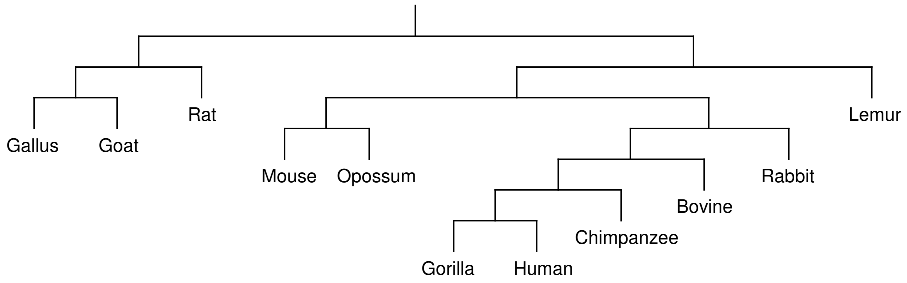

Fig. 10. The phylogenetic tree of the 11 species computed using Neighbor Joining algorithm applied on the distance matrix computed based on the GC content on the Symmetric Difference of the MAW sets (on RC setting).)

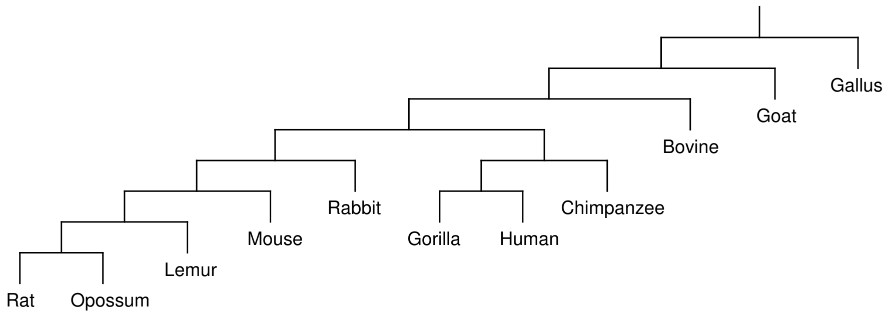

Fig. 11. The phylogenetic tree of the 11 species computed using UPGMA algorithm applied on the distance matrix computed based on the GC content on the RAW sets (on RC setting).)

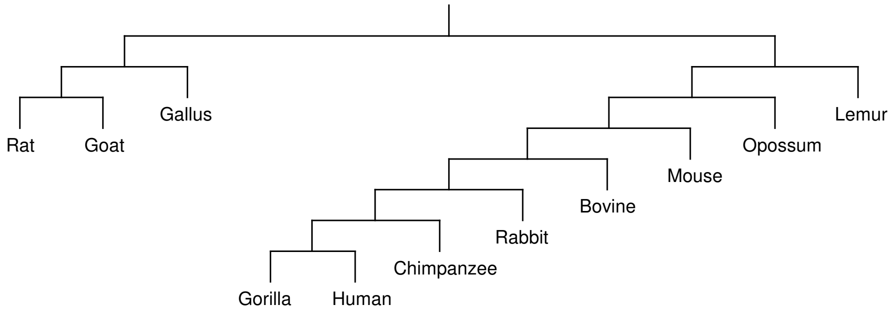

Fig. 12. The phylogenetic tree of the 11 species computed using Neighbor Joining algorithm applied on the distance matrix computed based on the GC content on the RAW sets (on RC setting).)

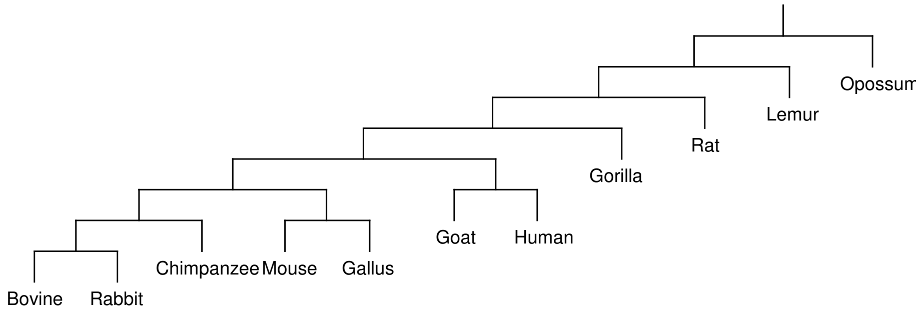

Fig. 13. The phylogenetic tree of the 11 species computed using UPGMA algorithm applied on the distance matrix computed based on the GC content on the Intersection of the MAW sets (on RC setting).)

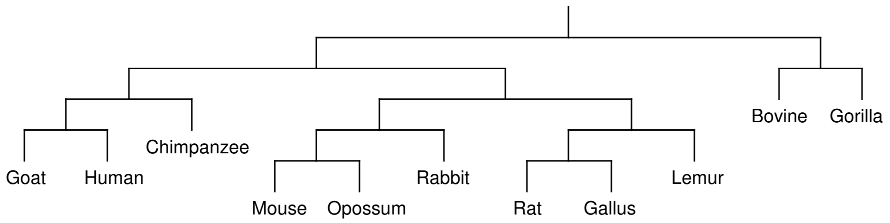

Fig. 14. The phylogenetic tree of the 11 species computed using Neighbor Joining algorithm applied on the distance matrix computed based on the GC content on the Intersection of the MAW sets (on RC setting).)

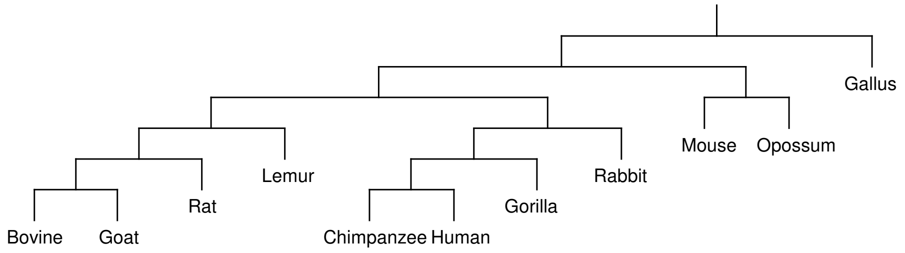

Fig. 15. The phylogenetic tree of the 11 species computed using UPGMA algorithm applied on the distance matrix computed based on the Total Variation Distance on the MAW sets (on RC setting).)

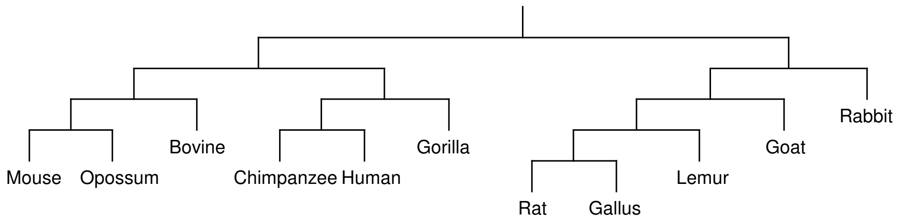

Fig. 16. The phylogenetic tree of the 11 species computed using Neighbor Joining algorithm applied on the distance matrix computed based on the Total Variation Distance on the MAW sets (on RC setting).)

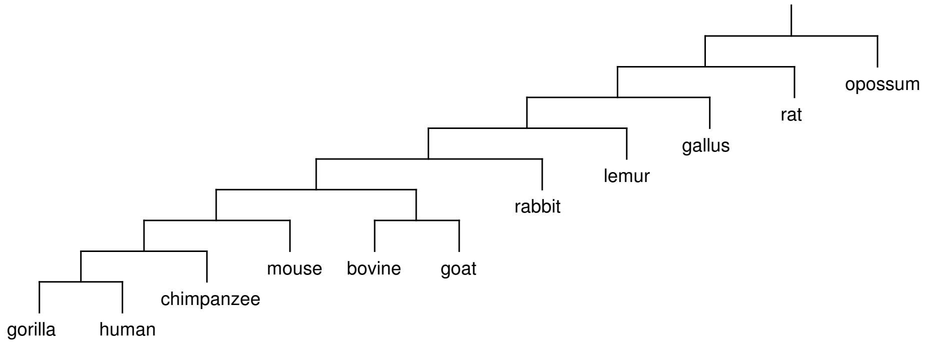

Fig. 17. The phylogenetic tree of the 11 species computed using UPGMA algorithm applied on the distance matrix computed based on the Length Weighted Index on the Symmetric Difference of the MAW sets (on NoRC setting).

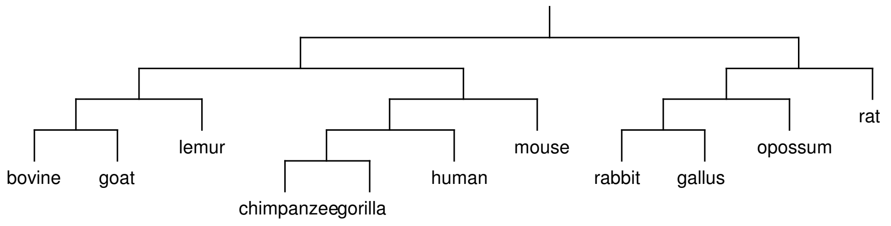

Fig. 18. The phylogenetic tree of the 11 species computed using Neighboring Joining algorithm applied on the distance matrix computed based on the Length Weighted Index on the Symmetric Difference of the MAW sets (on NoRC setting).

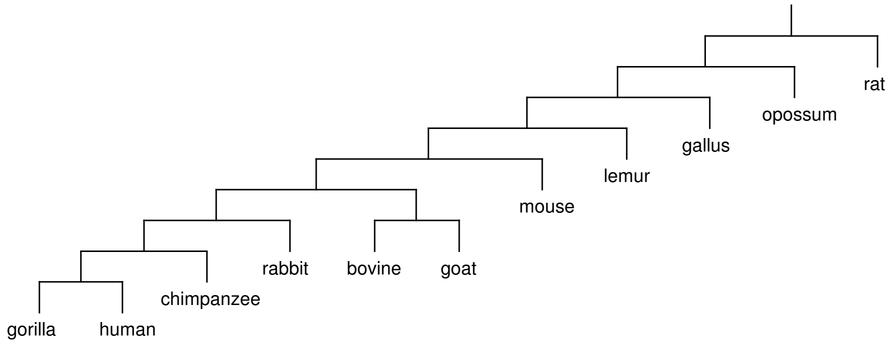

Fig. 19. The phylogenetic tree of the 11 species computed using UPGMA algorithm applied on the distance matrix computed based on the Length Weighted Index on the RAW sets (on NoRC setting).)

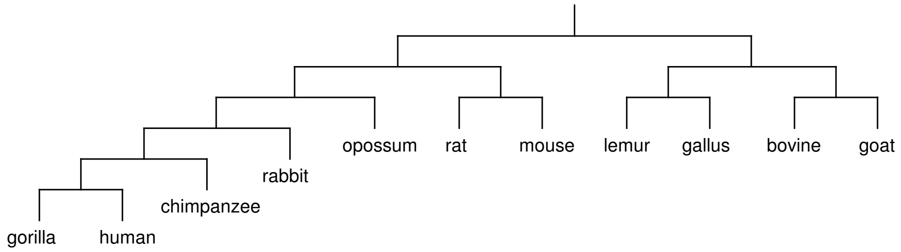

Fig. 20. The phylogenetic tree of the 11 species computed using Neighbor Joining algorithm applied on the distance matrix computed based on the Length Weighted Index on the RAW sets (on NoRC setting).)

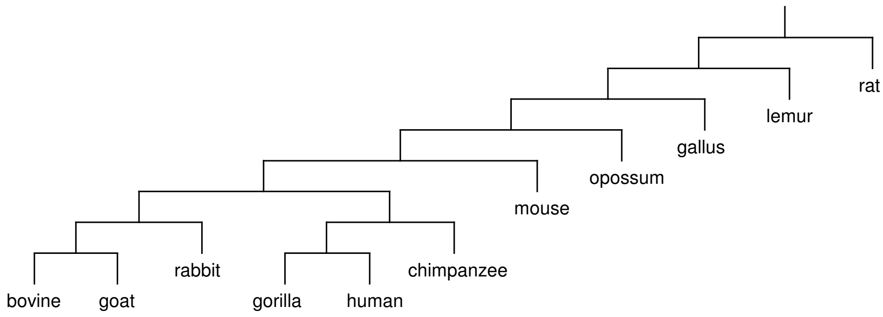

Fig. 21. The phylogenetic tree of the 11 species computed using UPGMA algorithm applied on the distance matrix computed based on the Length Weighted Index on the Intersections of the MAW sets (on NoRC setting).)

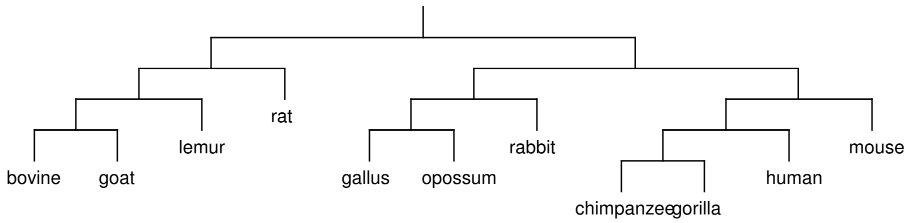

Fig. 22. The phylogenetic tree of the 11 species computed using Neighboring Joining algorithm applied on the distance matrix computed based on the Length Weighted Index on the Intersection of the MAW sets (on NoRC setting).)

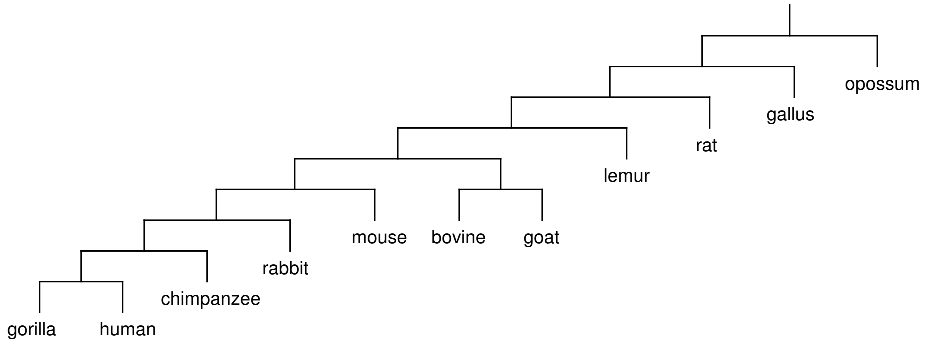

Fig. 23. The phylogenetic tree of the 11 species computed using UPGMA algorithm applied on the distance matrix computed based on the Jaccard Index on the MAW sets (on NoRC setting).)

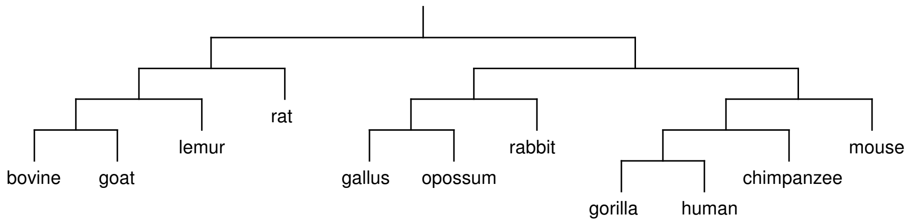

Fig. 24. The phylogenetic tree of the 11 species computed using Neighbor Joining algorithm applied on the distance matrix computed based on the Jaccard Index on the MAW sets (on NoRC setting).)

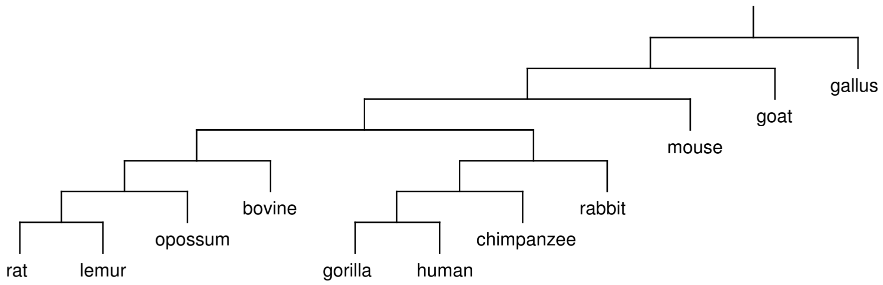

Fig. 25. The phylogenetic tree of the 11 species computed using UPGMA algorithm applied on the distance matrix computed based on the *GC* content on the Symmetric Difference of the MAW sets (on NoRC setting).)

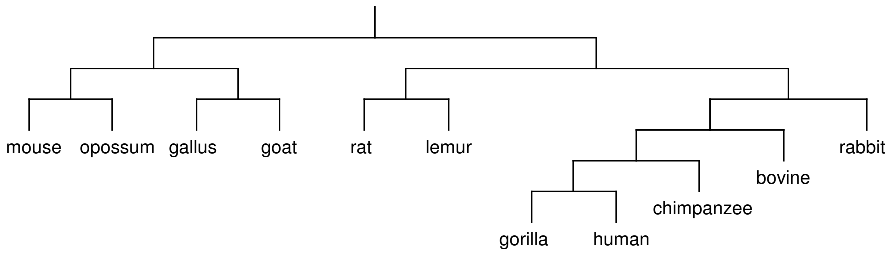

Fig. 26. The phylogenetic tree of the 11 species computed using Neighbor Joining algorithm applied on the distance matrix computed based on the *GC* content on the Symmetric Difference of the MAW sets (on NoRC setting).)

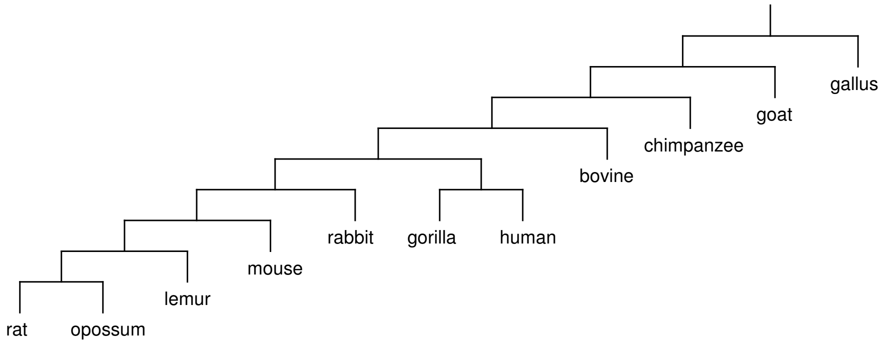

Fig. 27. The phylogenetic tree of the 11 species computed using UPGMA algorithm applied on the distance matrix computed based on the *GC* content on the RAW sets (on NoRC setting).)

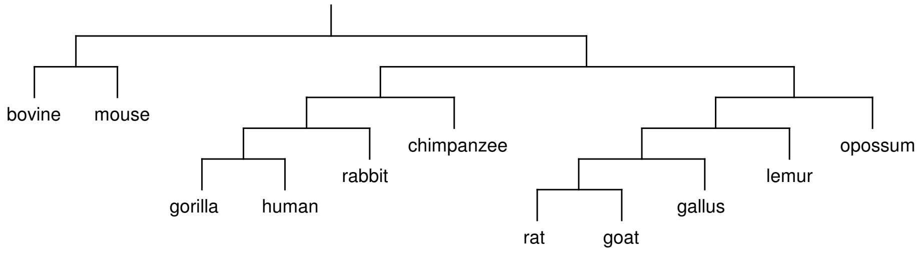

Fig. 28. The phylogenetic tree of the 11 species computed using Neighbor Joining algorithm applied on the distance matrix computed based on the GC content on the RAW sets (on NoRC setting).)

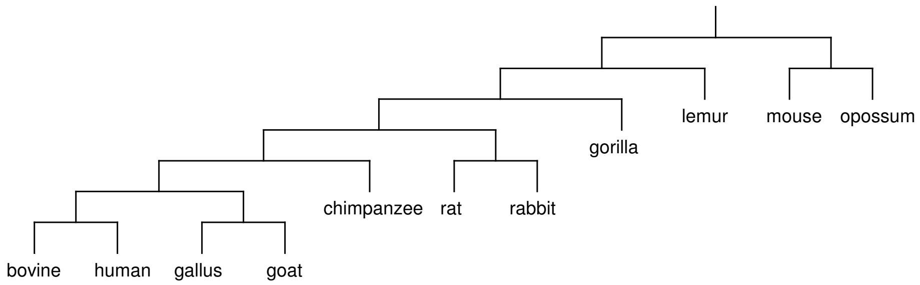

Fig. 29. The phylogenetic tree of the 11 species computed using UPGMA algorithm applied on the distance matrix computed based on the GC content on the Intersection of the MAW sets (on NoRC setting).)

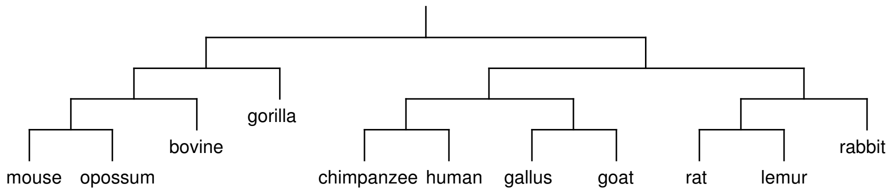

Fig. 30. The phylogenetic tree of the 11 species computed using Neighbor Joining algorithm applied on the distance matrix computed based on the *GC* content on the Intersection of the MAW sets (on NoRC setting).)

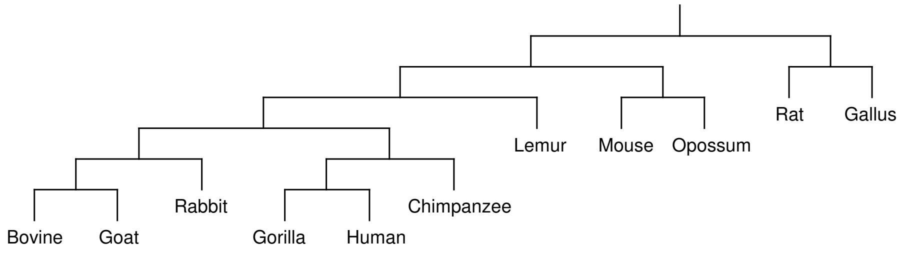

Fig. 31. The phylogenetic tree of the 11 species computed using UPGMA algorithm applied on the distance matrix computed based on the Total Variation Distance on the MAW sets (on NoRC setting).)

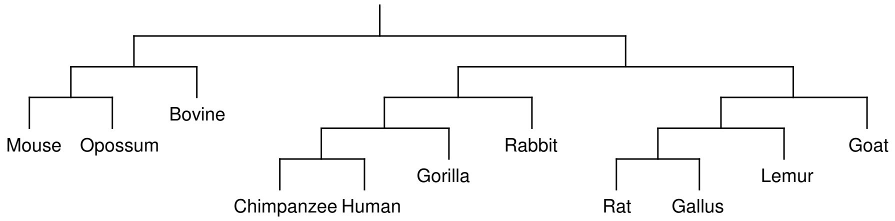

Fig. 32. The phylogenetic tree of the 11 species computed using Neighbor Joining algorithm applied on the distance matrix computed based on the Total Variation Distance on the MAW sets (on NoRC setting).)
