# Supplementary material for: Absent words and the (dis)similarity analysis of DNA sequences: an experimental study
Source: BMC Res Notes. 2016 Mar 22;9:186. doi: 10.1186/s13104-016-1972-z (PMC4804535; doi:10.1186/s13104-016-1972-z)
Supplement: Supplementary file 2 — 10.1186/s13104-016-1972-z All sorted difference tables. In this file (AllTables), for each distance matrix, a sorted list of each species from a particular species (left most column of each row) is provided. [file 13104_2016_1972_MOESM2_ESM.pdf]

Table 1. The distance matrix based on the GCC Index on Intersection of MAW sets (on RC setting).

Species	human	goat	opossum	gallus	lemur	mouse	rabbit	rat	gorilla	bovine	chimp
human		0.41	0.48	0.41	0.47	0.46	0.43	0.48	0.44	0.41	0.43
goat			0.46	0.40	0.47	0.45	0.41	0.45	0.42	0.45	0.42
opossum				0.46	0.52	0.44	0.43	0.51	0.48	0.43	0.48
gallus					0.41	0.39	0.42	0.39	0.40	0.40	0.41
lemur						0.46	0.44	0.46	0.48	0.45	0.48
mouse							0.41	0.46	0.46	0.44	0.46
rabbit								0.41	0.42	0.40	0.42
rat									0.47	0.43	0.47
gorilla										0.41	0.44
bovine											0.41
chimp											

Table 2. The distance matrix based on the GCC Index on RAW sets (on RC setting).

Species	human	goat	opossum	gallus	lemur	mouse	rabbit	rat	gorilla	bovine	chimp
human		0.58	0.54	0.60	0.55	0.55	0.55	0.55	0.50	0.56	0.50
goat			0.56	0.61	0.57	0.58	0.58	0.56	0.59	0.62	0.59
opossum				0.57	0.53	0.54	0.54	0.53	0.55	0.55	0.55
gallus					0.58	0.59	0.60	0.58	0.60	0.60	0.60
lemur						0.55	0.55	0.54	0.56	0.56	0.56
mouse							0.56	0.54	0.56	0.56	0.56
rabbit								0.55	0.56	0.57	0.56
rat									0.55	0.55	0.55
gorilla										0.58	0.59
bovine											0.58
chimp											

Table 3. The distance matrix based on the GCC Index on Symmetric Difference of MAW sets (on RC setting).

Species	human	goat	opossum	gallus	lemur	mouse	rabbit	rat	gorilla	bovine	chimp
human		0.53	0.54	0.56	0.54	0.56	0.53	0.54	0.40	0.52	0.40
goat			0.55	0.57	0.56	0.57	0.55	0.54	0.55	0.62	0.53
opossum				0.57	0.54	0.54	0.53	0.53	0.55	0.54	0.54
gallus					0.55	0.56	0.58	0.54	0.57	0.58	0.55
lemur						0.54	0.54	0.52	0.56	0.55	0.54
mouse							0.55	0.54	0.58	0.56	0.56
rabbit								0.52	0.53	0.55	0.52
rat									0.54	0.53	0.53
gorilla										0.53	0.41
bovine											0.52
chimp											

Table 4. The distance matrix based on the Jaccard Index on MAW sets (on RC setting).

Species	human	goat	opossum	gallus	lemur	mouse	rabbit	rat	gorilla	bovine	chimp
human		0.70	0.82	0.80	0.76	0.70	0.61	0.80	0.15	0.69	0.26
goat			0.84	0.74	0.74	0.77	0.77	0.79	0.69	0.36	0.71
opossum				0.85	0.87	0.91	0.84	0.90	0.82	0.85	0.82
gallus					0.81	0.82	0.79	0.85	0.80	0.81	0.80
lemur						0.83	0.81	0.81	0.76	0.72	0.77
mouse							0.78	0.78	0.64	0.74	0.68
rabbit								0.81	0.63	0.75	0.65
rat									0.80	0.82	0.82
gorilla										0.67	0.15
bovine											0.69
chimp											

Table 5. The distance matrix based on the Length Weighted Index on Intersection of MAW sets (on RC setting).

Species	human	goat	opossum	gallus	lemur	mouse	rabbit	rat	gorilla	bovine	chimp
human		-7.15	-4.63	-5.38	-6.91	-7.67	-7.99	-6.19	-13.21	-7.11	-12.55
goat			-4.29	-6.49	-6.81	-6.61	-6.34	-5.42	-7.24	-10.59	-7.14
opossum				-4.13	-4.44	-2.77	-4.12	-3.41	-4.64	-3.79	-4.76
gallus					-5.34	-5.33	-5.89	-3.96	-5.29	-5.21	-5.44
lemur						-5.84	-5.70	-5.82	-7.04	-7.40	-6.97
mouse							-5.92	-6.14	-8.27	-6.97	-7.74
rabbit								-5.49	-7.54	-6.35	-7.56
rat									-6.02	-5.21	-5.92
gorilla										-7.28	-13.36
bovine											-7.18
chimp											

Table 6. The distance matrix based on the Length Weighted Index on RAW sets (on RC setting).

Species	human	goat	opossum	gallus	lemur	mouse	rabbit	rat	gorilla	bovine	chimp
human		23.39	26.94	28.34	27.82	23.49	19.31	27.88	4.77	21.60	7.26
goat			28.71	24.16	25.89	25.52	24.33	27.43	21.77	8.73	24.26
opossum				29.55	31.23	29.21	26.69	30.52	26.90	28.16	28.44
gallus					28.66	30.22	26.27	30.89	28.25	26.21	30.51
lemur						30.21	27.63	30.96	27.77	25.91	30.27
mouse							24.09	26.43	20.98	23.17	23.29
rabbit								29.19	19.02	22.28	21.50
rat									28.37	27.95	30.21
gorilla										19.48	9.62
bovine											21.97
chimp											

Table 7. The distance matrix based on the Length Weighted Index on Symmetric Difference of MAW sets (on RC setting).

Species	human	goat	opossum	gallus	lemur	mouse	rabbit	rat	gorilla	bovine	chimp
human		12.79	16.83	17.17	15.46	12.95	11.65	17.10	1.95	13.08	4.35
goat			16.06	13.49	14.21	13.62	13.48	17.19	12.45	4.67	13.71
opossum				17.22	17.95	20.30	16.93	20.20	16.66	17.27	17.48
gallus					18.01	17.04	15.25	20.94	17.19	16.27	17.96
lemur						17.35	16.96	18.58	15.04	13.25	16.25
mouse							15.54	16.96	11.61	13.11	13.72
rabbit								17.58	12.40	13.68	13.41
rat									17.28	17.81	18.55
gorilla										12.57	2.56
bovine											13.84
chimp											

Table 8. The distance matrix based on the Total Variation Distance of MAW sets (on RC setting).

Species	human	goat	opossum	gallus	lemur	mouse	rabbit	rat	gorilla	bovine	chimp
human		0.09	0.11	0.17	0.09	0.13	0.08	0.12	0.04	0.06	0.03
goat			0.15	0.15	0.08	0.10	0.09	0.07	0.11	0.07	0.09
opossum				0.25	0.18	0.06	0.12	0.14	0.10	0.09	0.12
gallus					0.13	0.19	0.16	0.11	0.18	0.18	0.18
lemur						0.14	0.10	0.10	0.11	0.10	0.13
mouse							0.10	0.08	0.11	0.08	0.13
rabbit								0.10	0.07	0.09	0.10
rat									0.13	0.08	0.13
gorilla										0.08	0.06
bovine											0.07
chimp											

Table 9. The distance matrix based on the GCC Index on intersection of MAW sets (on NoRC setting).

Species	human	goat	opossum	gallus	lemur	mouse	rabbit	rat	gorilla	bovine	chimp
human		0.40	0.47	0.40	0.44	0.45	0.41	0.44	0.42	0.40	0.41
goat			0.49	0.34	0.47	0.47	0.39	0.44	0.42	0.45	0.41
opossum				0.46	0.51	0.44	0.45	0.49	0.46	0.43	0.45
gallus					0.41	0.37	0.43	0.37	0.40	0.36	0.41
lemur						0.45	0.42	0.42	0.46	0.46	0.45
mouse							0.44	0.46	0.47	0.45	0.47
rabbit								0.41	0.41	0.41	0.41
rat									0.44	0.42	0.43
gorilla										0.41	0.43
bovine											0.41
chimp											

Table 10. The distance matrix based on the GCC Index on RAW sets (on NoRC setting).

Species	human	goat	opossum	gallus	lemur	mouse	rabbit	rat	gorilla	bovine	chimp
human		0.58	0.54	0.59	0.55	0.55	0.55	0.55	0.50	0.56	0.52
goat			0.55	0.60	0.56	0.57	0.58	0.56	0.59	0.61	0.59
opossum				0.57	0.52	0.53	0.54	0.52	0.55	0.54	0.55
gallus					0.57	0.59	0.59	0.57	0.60	0.60	0.60
lemur						0.54	0.55	0.53	0.56	0.55	0.56
mouse							0.56	0.54	0.56	0.56	0.56
rabbit								0.55	0.56	0.57	0.57
rat									0.55	0.55	0.56
gorilla										0.57	0.62
bovine											0.58
chimp											

Table 11. The distance matrix based on the GCC Index on Symmetric Index of MAW sets (on NoRC setting).

Species	human	goat	opossum	gallus	lemur	mouse	rabbit	rat	gorilla	bovine	chimp
human		0.55	0.55	0.59	0.54	0.57	0.54	0.55	0.39	0.54	0.40
goat			0.56	0.58	0.56	0.59	0.56	0.56	0.57	0.63	0.55
opossum				0.58	0.53	0.54	0.54	0.53	0.55	0.54	0.54
gallus					0.57	0.58	0.60	0.57	0.59	0.58	0.58
lemur						0.54	0.54	0.52	0.55	0.55	0.54
mouse							0.57	0.56	0.60	0.57	0.58
rabbit								0.54	0.55	0.56	0.53
rat									0.55	0.54	0.54
gorilla										0.55	0.41
bovine											0.54
chimp											

Table 12. The distance matrix based on the Jaccard Index on MAW sets (on NoRC setting).

Species	human	goat	opossum	gallus	lemur	mouse	rabbit	rat	gorilla	bovine	chimp
human		0.73	0.85	0.84	0.81	0.72	0.68	0.82	0.14	0.71	0.26
goat			0.88	0.81	0.77	0.80	0.80	0.82	0.72	0.38	0.74
opossum				0.89	0.92	0.94	0.86	0.93	0.84	0.89	0.84
gallus					0.85	0.88	0.81	0.90	0.85	0.86	0.85
lemur						0.85	0.84	0.85	0.80	0.76	0.82
mouse							0.83	0.82	0.66	0.78	0.70
rabbit								0.85	0.69	0.80	0.70
rat									0.83	0.84	0.85
gorilla										0.69	0.16
bovine											0.72
chimp											

Table 13. The distance matrix based on the Length Weighted Index on intersection of MAW sets (on NoRC setting).

Species	human	goat	opossum	gallus	lemur	mouse	rabbit	rat	gorilla	bovine	chimp
human		-3.73	-2.35	-2.40	-3.16	-4.01	-3.77	-2.92	-7.85	-3.73	-7.35
goat			-1.96	-2.90	-3.48	-3.38	-3.19	-2.61	-3.89	-5.97	-3.78
opossum				-1.73	-1.54	-1.06	-2.11	-1.39	-2.41	-1.65	-2.41
gallus					-2.53	-2.26	-3.01	-1.52	-2.28	-2.33	-2.40
lemur						-2.85	-2.60	-2.62	-3.36	-3.54	-3.20
mouse							-2.67	-2.76	-4.42	-3.52	-4.16
rabbit								-2.53	-3.71	-2.91	-3.78
rat									-2.91	-2.44	-2.62
gorilla										-3.89	-7.87
bovine											-3.77
chimp											

Table 14. The distance matrix based on the Length Weighted Index on RAW sets (on NoRC setting).

Species	human	goat	opossum	gallus	lemur	mouse	rabbit	rat	gorilla	bovine	chimp
human		12.94	14.16	15.55	15.08	12.50	10.06	14.79	2.06	11.85	3.35
goat			16.08	12.84	13.68	13.08	13.26	15.34	11.69	4.32	13.18
opossum				16.61	17.03	15.56	13.86	16.33	14.18	15.57	14.90
gallus					15.48	15.95	13.93	17.10	15.21	14.03	16.75
lemur						15.53	14.73	16.56	14.85	13.43	16.19
mouse							12.41	14.34	11.27	11.82	12.54
rabbit								15.96	9.60	11.81	11.09
rat									14.86	15.46	15.86
gorilla										10.33	8.89
bovine											11.82
chimp											

Table 15. The distance matrix based on the Length Weighted Index on Symmetric Difference of MAW sets (on NoRC setting).

Species	human	goat	opossum	gallus	lemur	mouse	rabbit	rat	gorilla	bovine	chimp
human		8.34	10.55	11.18	10.61	8.21	8.44	10.54	1.09	8.22	2.65
goat			10.37	9.23	9.00	8.50	8.63	10.21	8.06	2.78	8.83
opossum				11.01	12.34	12.60	10.24	12.08	10.45	10.87	11.00
gallus					11.08	10.93	9.17	12.55	11.46	10.22	11.76
lemur						10.69	10.93	11.29	10.24	8.76	11.11
mouse							10.11	10.32	7.43	8.11	8.50
rabbit								10.53	8.58	9.06	9.00
rat									10.60	10.41	11.71
gorilla										7.93	1.64
bovine											8.71
chimp											

Table 16. The distance matrix based on the Total Variation Distance of MAW sets (on NoRC setting).

Species	human	goat	opossum	gallus	lemur	mouse	rabbit	rat	gorilla	bovine	chimp
human		0.09	0.12	0.18	0.09	0.14	0.08	0.14	0.03	0.06	0.03
goat			0.16	0.16	0.07	0.12	0.09	0.09	0.11	0.06	0.08
opossum				0.26	0.19	0.07	0.12	0.15	0.11	0.10	0.14
gallus					0.13	0.19	0.16	0.11	0.18	0.17	0.19
lemur						0.14	0.10	0.11	0.11	0.10	0.11
mouse							0.09	0.08	0.13	0.09	0.14
rabbit								0.10	0.07	0.07	0.09
rat									0.14	0.09	0.14
gorilla										0.08	0.06
bovine											0.07
chimp											
