# Supplementary material for: Absent words and the (dis)similarity analysis of DNA sequences: an experimental study
Source: BMC Res Notes. 2016 Mar 22;9:186. doi: 10.1186/s13104-016-1972-z (PMC4804535; doi:10.1186/s13104-016-1972-z)
Supplement: Supplementary file 3 — 10.1186/s13104-016-1972-z All phylogenetic trees. In this file (AllTree), all Phylogenetic trees computed based on the distance matrixes are provide. From each distance matrix, teo Phylogenetic trees are reconstructed using two algorithms, namely, UPGMA and NJ. [file 13104_2016_1972_MOESM3_ESM.pdf]

Table 1. The sorted list of each species from a particular species (left most column of each row) according to the computed distance based on the GCC Index on Intersection of MAW sets (on RC setting).

human	→bovine	→gallus	→goat	→rabbit	→chimp	→gorilla	→mouse	→lemur	→rat	→opossum
goat	→gallus	→rabbit	→human	→chimp	→gorilla	→bovine	→mouse	→rat	→opossum	→lemur
opossum	→bovine	→rabbit	→mouse	→gallus	→goat	→gorilla	→chimp	→human	→rat	→lemur
gallus	→mouse	→rat	→goat	→bovine	→gorilla	→chimp	→human	→lemur	→rabbit	→opossum
lemur	→gallus	→rabbit	→bovine	→mouse	→rat	→human	→goat	→chimp	→gorilla	→opossum
mouse	→gallus	→rabbit	→bovine	→opossum	→goat	→human	→lemur	→chimp	→rat	→gorilla
rabbit	→bovine	→goat	→rat	→mouse	→gallus	→chimp	→gorilla	→human	→opossum	→lemur
rat	→gallus	→rabbit	→bovine	→goat	→mouse	→lemur	→gorilla	→chimp	→human	→opossum
gorilla	→gallus	→bovine	→rabbit	→goat	→human	→chimp	→mouse	→rat	→opossum	→lemur
bovine	→gallus	→rabbit	→human	→chimp	→gorilla	→opossum	→rat	→mouse	→goat	→lemur
chimp	→gallus	→bovine	→rabbit	→goat	→human	→gorilla	→mouse	→rat	→lemur	→opossum

Table 2. The sorted list of each species from a particular species (left most column of each row) according to the computed distance based on the GCC Index on RAW sets (on RC setting).

human	→gorilla	→chimp	→opossum	→rat	→lemur	→rabbit	→mouse	→bovine	→goat	→gallus
goat	→rat	→opossum	→lemur	→human	→mouse	→rabbit	→chimp	→gorilla	→gallus	→bovine
opossum	→rat	→lemur	→mouse	→human	→rabbit	→bovine	→chimp	→gorilla	→goat	→gallus
gallus	→opossum	→rat	→lemur	→mouse	→human	→rabbit	→bovine	→chimp	→gorilla	→goat
lemur	→opossum	→rat	→mouse	→human	→rabbit	→chimp	→gorilla	→bovine	→goat	→gallus
mouse	→opossum	→rat	→lemur	→human	→rabbit	→chimp	→gorilla	→bovine	→goat	→gallus
rabbit	→opossum	→rat	→human	→lemur	→mouse	→chimp	→gorilla	→bovine	→goat	→gallus
rat	→opossum	→lemur	→mouse	→human	→rabbit	→bovine	→chimp	→gorilla	→goat	→gallus
gorilla	→human	→opossum	→rat	→lemur	→mouse	→rabbit	→bovine	→goat	→chimp	→gallus
bovine	→opossum	→rat	→human	→lemur	→mouse	→rabbit	→chimp	→gorilla	→gallus	→goat
chimp	→human	→opossum	→rat	→lemur	→mouse	→rabbit	→bovine	→goat	→gorilla	→gallus

Table 3. The sorted list of each species from a particular species (left most column of each row) according to the computed distance based on the GCC Index on Symmetric Difference of MAW sets (on RC setting).

human	→chimp	→gorilla	→bovine	→rabbit	→goat	→rat	→lemur	→opossum	→mouse	→gallus
goat	→chimp	→human	→rat	→gorilla	→opossum	→rabbit	→lemur	→mouse	→gallus	→bovine
opossum	→rat	→rabbit	→bovine	→chimp	→mouse	→lemur	→human	→gorilla	→goat	→gallus
gallus	→rat	→lemur	→chimp	→human	→mouse	→gorilla	→opossum	→goat	→bovine	→rabbit
lemur	→rat	→opossum	→rabbit	→human	→chimp	→mouse	→bovine	→gallus	→gorilla	→goat
mouse	→opossum	→rat	→lemur	→rabbit	→chimp	→human	→bovine	→gallus	→goat	→gorilla
rabbit	→chimp	→rat	→human	→gorilla	→opossum	→lemur	→bovine	→mouse	→goat	→gallus
rat	→lemur	→rabbit	→opossum	→chimp	→bovine	→human	→mouse	→gorilla	→goat	→gallus
gorilla	→human	→chimp	→rabbit	→bovine	→rat	→opossum	→goat	→lemur	→gallus	→mouse
bovine	→chimp	→human	→rat	→gorilla	→opossum	→rabbit	→lemur	→mouse	→gallus	→goat
chimp	→human	→gorilla	→rabbit	→bovine	→goat	→rat	→opossum	→lemur	→gallus	→mouse

Table 4. The sorted list of each species from a particular species (left most column of each row) according to the computed distance based on the Jaccard Index on MAW sets (on RC setting).

human	→gorilla	→chimp	→rabbit	→bovine	→mouse	→goat	→lemur	→gallus	→rat	→opossum
goat	→bovine	→gorilla	→human	→chimp	→lemur	→gallus	→rabbit	→mouse	→rat	→opossum
opossum	→chimp	→human	→gorilla	→rabbit	→goat	→gallus	→bovine	→lemur	→rat	→mouse
gallus	→goat	→rabbit	→human	→gorilla	→chimp	→bovine	→lemur	→mouse	→opossum	→rat
lemur	→bovine	→goat	→gorilla	→human	→chimp	→rabbit	→rat	→gallus	→mouse	→opossum
mouse	→gorilla	→chimp	→human	→bovine	→goat	→rat	→rabbit	→gallus	→lemur	→opossum
rabbit	→human	→gorilla	→chimp	→bovine	→goat	→mouse	→gallus	→lemur	→rat	→opossum
rat	→mouse	→goat	→human	→gorilla	→rabbit	→lemur	→chimp	→bovine	→gallus	→opossum
gorilla	→human	→chimp	→rabbit	→mouse	→bovine	→goat	→lemur	→gallus	→rat	→opossum
bovine	→goat	→gorilla	→human	→chimp	→lemur	→mouse	→rabbit	→gallus	→rat	→opossum
chimp	→gorilla	→human	→rabbit	→mouse	→bovine	→goat	→lemur	→gallus	→opossum	→rat

Table 5. The sorted list of each species from a particular species (left most column of each row) according to the computed distance based on the Length Weighted Index on Intersection of MAW sets (on RC setting).

human	→gorilla	→chimp	→rabbit	→mouse	→goat	→bovine	→lemur	→rat	→gallus	→opossum
goat	→bovine	→gorilla	→human	→chimp	→lemur	→mouse	→gallus	→rabbit	→rat	→opossum
opossum	→chimp	→gorilla	→human	→lemur	→goat	→gallus	→rabbit	→bovine	→rat	→mouse
gallus	→goat	→rabbit	→chimp	→human	→lemur	→mouse	→gorilla	→bovine	→opossum	→rat
lemur	→bovine	→gorilla	→chimp	→human	→goat	→mouse	→rat	→rabbit	→gallus	→opossum
mouse	→gorilla	→chimp	→human	→bovine	→goat	→rat	→rabbit	→lemur	→gallus	→opossum
rabbit	→human	→chimp	→gorilla	→bovine	→goat	→mouse	→gallus	→lemur	→rat	→opossum
rat	→human	→mouse	→gorilla	→chimp	→lemur	→rabbit	→goat	→bovine	→gallus	→opossum
gorilla	→chimp	→human	→mouse	→rabbit	→bovine	→goat	→lemur	→rat	→gallus	→opossum
bovine	→goat	→lemur	→gorilla	→chimp	→human	→mouse	→rabbit	→gallus	→rat	→opossum
chimp	→gorilla	→human	→mouse	→rabbit	→bovine	→goat	→lemur	→rat	→gallus	→opossum

Table 6. The sorted list of each species from a particular species (left most column of each row) according to the computed distance based on the Length Weighted Index on RAW sets (on RC setting).

human	→gorilla	→chimp	→rabbit	→bovine	→goat	→mouse	→opossum	→lemur	→rat	→gallus
goat	→bovine	→gorilla	→human	→gallus	→chimp	→rabbit	→mouse	→lemur	→rat	→opossum
opossum	→rabbit	→gorilla	→human	→bovine	→chimp	→goat	→mouse	→gallus	→rat	→lemur
gallus	→goat	→bovine	→rabbit	→gorilla	→human	→lemur	→opossum	→mouse	→chimp	→rat
lemur	→goat	→bovine	→rabbit	→gorilla	→human	→gallus	→mouse	→chimp	→rat	→opossum
mouse	→gorilla	→bovine	→chimp	→human	→rabbit	→goat	→rat	→opossum	→lemur	→gallus
rabbit	→gorilla	→human	→chimp	→bovine	→mouse	→goat	→gallus	→opossum	→lemur	→rat
rat	→mouse	→goat	→human	→bovine	→gorilla	→rabbit	→chimp	→opossum	→gallus	→lemur
gorilla	→human	→chimp	→rabbit	→bovine	→mouse	→goat	→opossum	→lemur	→gallus	→rat
bovine	→goat	→gorilla	→human	→chimp	→rabbit	→mouse	→lemur	→gallus	→rat	→opossum
chimp	→human	→gorilla	→rabbit	→bovine	→mouse	→goat	→opossum	→rat	→lemur	→gallus

Table 7. The sorted list of each species from a particular species (left most column of each row) according to the computed distance based on the Length Weighted Index on Symmetric Difference of MAW sets (on RC setting).

human	→gorilla	→chimp	→rabbit	→goat	→mouse	→bovine	→lemur	→opossum	→rat	→gallus
goat	→bovine	→gorilla	→human	→rabbit	→gallus	→mouse	→chimp	→lemur	→opossum	→rat
opossum	→goat	→gorilla	→human	→rabbit	→gallus	→bovine	→chimp	→lemur	→rat	→mouse
gallus	→goat	→rabbit	→bovine	→mouse	→human	→gorilla	→opossum	→chimp	→lemur	→rat
lemur	→bovine	→goat	→gorilla	→human	→chimp	→rabbit	→mouse	→opossum	→gallus	→rat
mouse	→gorilla	→human	→bovine	→goat	→chimp	→rabbit	→rat	→gallus	→lemur	→opossum
rabbit	→human	→gorilla	→chimp	→goat	→bovine	→gallus	→mouse	→opossum	→lemur	→rat
rat	→mouse	→human	→goat	→gorilla	→rabbit	→bovine	→chimp	→lemur	→opossum	→gallus
gorilla	→human	→chimp	→mouse	→rabbit	→goat	→bovine	→lemur	→opossum	→gallus	→rat
bovine	→goat	→gorilla	→human	→mouse	→lemur	→rabbit	→chimp	→gallus	→opossum	→rat
chimp	→gorilla	→human	→rabbit	→goat	→mouse	→bovine	→lemur	→opossum	→gallus	→rat

Table 8. The sorted list of each species from a particular species (left most column of each row) according to the computed distance based on the Total Variation Distance of MAW sets (on RC setting).

human	→chimp	→gorilla	→bovine	→rabbit	→goat	→lemur	→opossum	→rat	→mouse	→gallus
goat	→bovine	→rat	→lemur	→human	→chimp	→rabbit	→mouse	→gorilla	→gallus	→opossum
opossum	→mouse	→bovine	→gorilla	→human	→rabbit	→chimp	→rat	→goat	→lemur	→gallus
gallus	→rat	→lemur	→goat	→rabbit	→human	→bovine	→chimp	→gorilla	→mouse	→opossum
lemur	→goat	→human	→rabbit	→rat	→bovine	→gorilla	→gallus	→chimp	→mouse	→opossum
mouse	→opossum	→rat	→bovine	→rabbit	→goat	→gorilla	→human	→chimp	→lemur	→gallus
rabbit	→gorilla	→human	→bovine	→goat	→mouse	→lemur	→chimp	→rat	→opossum	→gallus
rat	→goat	→bovine	→mouse	→lemur	→rabbit	→gallus	→human	→chimp	→gorilla	→opossum
gorilla	→human	→chimp	→rabbit	→bovine	→opossum	→lemur	→goat	→mouse	→rat	→gallus
bovine	→human	→goat	→chimp	→rat	→mouse	→gorilla	→opossum	→rabbit	→lemur	→gallus
chimp	→human	→gorilla	→bovine	→goat	→rabbit	→opossum	→rat	→lemur	→mouse	→gallus

Table 9. The sorted list of each species from a particular species (left most column of each row) according to the computed distance based on the GCC Index on intersection of MAW sets (on NoRC setting).

human	→goat	→gallus	→bovine	→rabbit	→chimp	→gorilla	→lemur	→rat	→mouse	→opossum
goat	→gallus	→rabbit	→human	→chimp	→gorilla	→rat	→bovine	→lemur	→mouse	→opossum
opossum	→bovine	→mouse	→rabbit	→chimp	→gallus	→gorilla	→human	→goat	→rat	→lemur
gallus	→goat	→bovine	→mouse	→rat	→human	→gorilla	→lemur	→chimp	→rabbit	→opossum
lemur	→gallus	→rabbit	→rat	→human	→mouse	→chimp	→gorilla	→bovine	→goat	→opossum
mouse	→gallus	→opossum	→rabbit	→human	→lemur	→bovine	→rat	→goat	→gorilla	→chimp
rabbit	→goat	→human	→rat	→gorilla	→bovine	→chimp	→lemur	→gallus	→mouse	→opossum
rat	→gallus	→rabbit	→lemur	→bovine	→chimp	→human	→goat	→gorilla	→mouse	→opossum
gorilla	→gallus	→rabbit	→bovine	→human	→goat	→chimp	→rat	→opossum	→lemur	→mouse
bovine	→gallus	→human	→rabbit	→gorilla	→chimp	→rat	→opossum	→goat	→mouse	→lemur
chimp	→human	→goat	→gallus	→rabbit	→bovine	→rat	→gorilla	→opossum	→lemur	→mouse

Table 10. The sorted list of each species from a particular species (left most column of each row) according to the computed distance based on the GCC Index on RAW sets (on NoRC setting).

human	→gorilla	→chimp	→opossum	→rat	→lemur	→mouse	→rabbit	→bovine	→goat	→gallus
goat	→opossum	→rat	→lemur	→mouse	→human	→rabbit	→gorilla	→chimp	→gallus	→bovine
opossum	→rat	→lemur	→mouse	→human	→rabbit	→bovine	→gorilla	→chimp	→goat	→gallus
gallus	→opossum	→rat	→lemur	→mouse	→human	→rabbit	→bovine	→gorilla	→chimp	→goat
lemur	→opossum	→rat	→mouse	→human	→rabbit	→bovine	→gorilla	→chimp	→goat	→gallus
mouse	→opossum	→rat	→lemur	→human	→rabbit	→bovine	→gorilla	→chimp	→goat	→gallus
rabbit	→opossum	→rat	→lemur	→human	→mouse	→gorilla	→bovine	→chimp	→goat	→gallus
rat	→opossum	→lemur	→mouse	→human	→rabbit	→bovine	→gorilla	→goat	→chimp	→gallus
gorilla	→human	→opossum	→rat	→lemur	→mouse	→rabbit	→bovine	→goat	→gallus	→chimp
bovine	→opossum	→rat	→lemur	→mouse	→human	→rabbit	→gorilla	→chimp	→gallus	→goat
chimp	→human	→opossum	→rat	→lemur	→mouse	→rabbit	→bovine	→goat	→gallus	→gorilla

Table 11. The sorted list of each species from a particular species (left most column of each row) according to the computed distance based on the GCC Index on Symmetric Index of MAW sets (on NoRC setting).

human	→gorilla	→chimp	→lemur	→rabbit	→bovine	→goat	→opossum	→rat	→mouse	→gallus
goat	→human	→chimp	→opossum	→lemur	→rabbit	→rat	→gorilla	→gallus	→mouse	→bovine
opossum	→lemur	→rat	→mouse	→rabbit	→bovine	→chimp	→human	→gorilla	→goat	→gallus
gallus	→lemur	→rat	→goat	→opossum	→mouse	→bovine	→chimp	→human	→gorilla	→rabbit
lemur	→rat	→opossum	→human	→mouse	→rabbit	→chimp	→gorilla	→bovine	→goat	→gallus
mouse	→opossum	→lemur	→rat	→human	→rabbit	→bovine	→gallus	→chimp	→goat	→gorilla
rabbit	→chimp	→human	→opossum	→lemur	→rat	→gorilla	→goat	→bovine	→mouse	→gallus
rat	→lemur	→opossum	→rabbit	→bovine	→chimp	→human	→gorilla	→goat	→mouse	→gallus
gorilla	→human	→chimp	→opossum	→lemur	→rabbit	→rat	→bovine	→goat	→gallus	→mouse
bovine	→human	→opossum	→rat	→chimp	→lemur	→gorilla	→rabbit	→mouse	→gallus	→goat
chimp	→human	→gorilla	→rabbit	→opossum	→lemur	→rat	→bovine	→goat	→gallus	→mouse

Table 12. The sorted list of each species from a particular species (left most column of each row) according to the computed distance based on the Jaccard Index on MAW sets (on NoRC setting).

human	→gorilla	→chimp	→rabbit	→bovine	→mouse	→goat	→lemur	→rat	→gallus	→opossum
goat	→bovine	→gorilla	→human	→chimp	→lemur	→mouse	→rabbit	→gallus	→rat	→opossum
opossum	→gorilla	→chimp	→human	→rabbit	→goat	→gallus	→bovine	→lemur	→rat	→mouse
gallus	→goat	→rabbit	→human	→lemur	→gorilla	→chimp	→bovine	→mouse	→opossum	→rat
lemur	→bovine	→goat	→gorilla	→human	→chimp	→rabbit	→gallus	→mouse	→rat	→opossum
mouse	→gorilla	→chimp	→human	→bovine	→goat	→rat	→rabbit	→lemur	→gallus	→opossum
rabbit	→human	→gorilla	→chimp	→goat	→bovine	→gallus	→mouse	→lemur	→rat	→opossum
rat	→human	→goat	→mouse	→gorilla	→bovine	→lemur	→rabbit	→chimp	→gallus	→opossum
gorilla	→human	→chimp	→mouse	→rabbit	→bovine	→goat	→lemur	→rat	→opossum	→gallus
bovine	→goat	→gorilla	→human	→chimp	→lemur	→mouse	→rabbit	→rat	→gallus	→opossum
chimp	→gorilla	→human	→mouse	→rabbit	→bovine	→goat	→lemur	→opossum	→gallus	→rat

Table 13. The sorted list of each species from a particular species (left most column of each row) according to the computed distance based on the Length Weighted Index on intersection of MAW sets (on NoRC setting).

human	→gorilla	→chimp	→mouse	→rabbit	→goat	→bovine	→lemur	→rat	→gallus	→opossum
goat	→bovine	→gorilla	→chimp	→human	→lemur	→mouse	→rabbit	→gallus	→rat	→opossum
opossum	→gorilla	→chimp	→human	→rabbit	→goat	→gallus	→bovine	→lemur	→rat	→mouse
gallus	→rabbit	→goat	→lemur	→human	→chimp	→bovine	→gorilla	→mouse	→opossum	→rat
lemur	→bovine	→goat	→gorilla	→chimp	→human	→mouse	→rat	→rabbit	→gallus	→opossum
mouse	→gorilla	→chimp	→human	→bovine	→goat	→lemur	→rat	→rabbit	→gallus	→opossum
rabbit	→chimp	→human	→gorilla	→goat	→gallus	→bovine	→mouse	→lemur	→rat	→opossum
rat	→human	→gorilla	→mouse	→lemur	→chimp	→goat	→rabbit	→bovine	→gallus	→opossum
gorilla	→chimp	→human	→mouse	→goat	→bovine	→rabbit	→lemur	→rat	→opossum	→gallus
bovine	→goat	→gorilla	→chimp	→human	→lemur	→mouse	→rabbit	→rat	→gallus	→opossum
chimp	→gorilla	→human	→mouse	→goat	→rabbit	→bovine	→lemur	→rat	→opossum	→gallus

Table 14. The sorted list of each species from a particular species (left most column of each row) according to the computed distance based on the Length Weighted Index on RAW sets (on NoRC setting).

human	→gorilla	→chimp	→rabbit	→bovine	→mouse	→goat	→opossum	→rat	→lemur	→gallus
goat	→bovine	→gorilla	→gallus	→human	→mouse	→chimp	→rabbit	→lemur	→rat	→opossum
opossum	→rabbit	→human	→gorilla	→chimp	→mouse	→bovine	→goat	→rat	→gallus	→lemur
gallus	→goat	→rabbit	→bovine	→gorilla	→lemur	→human	→mouse	→opossum	→chimp	→rat
lemur	→bovine	→goat	→rabbit	→gorilla	→human	→gallus	→mouse	→chimp	→rat	→opossum
mouse	→gorilla	→bovine	→rabbit	→human	→chimp	→goat	→rat	→lemur	→opossum	→gallus
rabbit	→gorilla	→human	→chimp	→bovine	→mouse	→goat	→opossum	→gallus	→lemur	→rat
rat	→mouse	→human	→gorilla	→goat	→bovine	→chimp	→rabbit	→opossum	→lemur	→gallus
gorilla	→human	→chimp	→rabbit	→bovine	→mouse	→goat	→opossum	→lemur	→rat	→gallus
bovine	→goat	→gorilla	→rabbit	→chimp	→mouse	→human	→lemur	→gallus	→rat	→opossum
chimp	→human	→gorilla	→rabbit	→bovine	→mouse	→goat	→opossum	→rat	→lemur	→gallus

Table 15. The sorted list of each species from a particular species (left most column of each row) according to the computed distance based on the Length Weighted Index on Symmetric Difference of MAW sets (on NoRC setting).

human	→gorilla	→chimp	→mouse	→bovine	→goat	→rabbit	→rat	→opossum	→lemur	→gallus
goat	→bovine	→gorilla	→human	→mouse	→rabbit	→chimp	→lemur	→gallus	→rat	→opossum
opossum	→rabbit	→goat	→gorilla	→human	→bovine	→chimp	→gallus	→rat	→lemur	→mouse
gallus	→rabbit	→goat	→bovine	→mouse	→opossum	→lemur	→human	→gorilla	→chimp	→rat
lemur	→bovine	→goat	→gorilla	→human	→mouse	→rabbit	→gallus	→chimp	→rat	→opossum
mouse	→gorilla	→bovine	→human	→goat	→chimp	→rabbit	→rat	→lemur	→gallus	→opossum
rabbit	→human	→gorilla	→goat	→chimp	→bovine	→gallus	→mouse	→opossum	→rat	→lemur
rat	→goat	→mouse	→bovine	→rabbit	→human	→gorilla	→lemur	→chimp	→opossum	→gallus
gorilla	→human	→chimp	→mouse	→bovine	→goat	→rabbit	→lemur	→opossum	→rat	→gallus
bovine	→goat	→gorilla	→mouse	→human	→chimp	→lemur	→rabbit	→gallus	→rat	→opossum
chimp	→gorilla	→human	→mouse	→bovine	→goat	→rabbit	→opossum	→lemur	→rat	→gallus

Table 16. The sorted list of each species from a particular species (left most column of each row) according to the computed distance based on the Total Variation Distance of MAW sets (on NoRC setting).

human	→gorilla	→chimp	→bovine	→rabbit	→lemur	→goat	→opossum	→mouse	→rat	→gallus
goat	→bovine	→lemur	→chimp	→rabbit	→human	→rat	→gorilla	→mouse	→gallus	→opossum
opossum	→mouse	→bovine	→gorilla	→human	→rabbit	→chimp	→rat	→goat	→lemur	→gallus
gallus	→rat	→lemur	→goat	→rabbit	→bovine	→gorilla	→human	→mouse	→chimp	→opossum
lemur	→goat	→human	→rabbit	→bovine	→rat	→chimp	→gorilla	→gallus	→mouse	→opossum
mouse	→opossum	→rat	→bovine	→rabbit	→goat	→gorilla	→lemur	→human	→chimp	→gallus
rabbit	→bovine	→gorilla	→human	→goat	→mouse	→chimp	→lemur	→rat	→opossum	→gallus
rat	→mouse	→bovine	→goat	→rabbit	→lemur	→gallus	→gorilla	→chimp	→human	→opossum
gorilla	→human	→chimp	→rabbit	→bovine	→lemur	→goat	→opossum	→mouse	→rat	→gallus
bovine	→human	→goat	→rabbit	→chimp	→gorilla	→mouse	→rat	→opossum	→lemur	→gallus
chimp	→human	→gorilla	→bovine	→goat	→rabbit	→lemur	→rat	→opossum	→mouse	→gallus
